# The Work of the Hospitals Association

**Published:** 1893-08-12

**Authors:** 


### The Hospital

AN INSTITUTIONAL JOURNAL OF

### Science, Medicine, Nursing, and Philanthropy.

*For Hospitals, Asylums, Infirmaries, Dispensaries, Medical Practitioners, Students, Nurses, and the Charitable Public.*

VOL. XIV.—No. 359.] AUG. 12, 1893

### THE WORK OF THE HOSPITALS ASSOCIATION

AN Association that at the end of its ninth year is able to report that most of the objects which it has striven to promote have been attained is well entitled to congratulation. This is the fortunate position which the Hospitals Association now occupies. The Association was formed in February, 1884. To a number of the friends of hospitals it was borne home that although the hospitals of this country and of the Metropolis in particular have many common interests and common needs there was no Round Table at which those engaged in hospital work might take counsel, no platform from which the common needs of hospitals might be pressed. The Hospitals Association is the outcome of the conviction on the part of those who founded it that for the sake of the hospital system of this country it was very desirable that a body should be called into being which should enable hospital workers to come together, make available the best knowledge on hospital questions of all kinds, and voice well considered opinion on all hospital interests.

To what extent have the hopes of those who founded the Association and who have maintained it been realised? The history of the Association is written in the reports that have been issued from year to year. Although at the outset the Association had its own share of the difficulties that seem to be inseparable from new movements, these reports record steady progress. Each is more gratifying than that which has preceded it, and the report for 1892, read at the ninth annual meeting held at St. Thomas's Hospital on Friday, the 4th of this month, is in more than one respect the most gratifying of all. From the very first the Association did good in that it afforded those connected with hospitals an opportunity of meeting and of discussing hospital questions. The schedule of papers that have been read before the Association from its foundation shows that almost all the subjects that are of first importance to British hospitals have been treated by competent authorities, and discussed by the members of the Association. Latterly there have been fewer meetings and papers, the Council being of opinion that the general sense of the members is against the undue multiplication of meetings. This is not only wise in itself, but it shows that the work of the Association in promoting co-operation between those connected with hospitals has been so thoroughly done that frequent meetings are no longer necessary to maintain this co operation. The report that was submitted the other day mentions "the promotion of co operation between managers of existing institutions" as one of the great objects of the Association that has been achieved, and under this head it goes on to say, "There never was a time when the managers of hospitals throughout the country, and, indeed, in all English-speaking countries too, were more disposed than at present to co-operate with one another. This fact was abundantly apparent at the recent congress held at Chicago. It finds further illustration in the fact that every hospital board-room in London is now open to the Council of the Association for the purposes of its meetings, and that hospital secretaries have associated themselves together in a club, where they successfully combine business with pleasure." This in itself is a result of which the Association may well be proud, and on account of which it deserves the thanks of all who have the welfare of hospitals at heart.

The deliberations of the Association bore early fruit in common action with a view to improving the hospital system of this country. Legislation affecting hospitals was carefully watched, and the Association was instrumental in getting more than one useful change effected in the laws bearing upon medical charities. From the very first the efforts of the Association were directed towards two objects that were deemed pre-eminently desirable in the interests of British hospitals and of the British public. One of these was the adoption by the hospitals of this country of a uniform system of accounts, the other the appointment of a Royal Commission to inquire into the circumstances, conduct, and needs of the medical charities. Both of these things have been attained. Thanks to the Council of the Metropolitan Hospital Sunday Fund which is gradually making the keeping of accounts on a uniform system a condition of grants, "every well managed hospital in London now keeps its accounts on the same system." Persistent representations to Government and other authorities had their reward in the appointment of the Lords Committee on Metropolitan Medical Charities, which collected a great deal of information and issued an important report. One of the recommendations of the Lords Committee was the creation of a Central Hospitals Board for London. It is not yet quite certain whether this Board will be called into being, as the Committee to whom the matter has been referred has not yet reported. It is to be hoped that a decision will be arrived at one way or the other with the least possible delay.

But the Association has done more than voice hospital opinion on these and other questions. It has been the means of bringing into existence two agencies that have already accomplished a great deal of good, and that will do even greater good in the future. The Royal National Pension Fund for Nurses, founded in 1887, is a child of the Hospitals Association. There is no need to dilate here upon the remarkable success which has attended this scheme. Another project carried to a successful issue is one that has enabled the Association to become the benefactor of the Metropolis at large. A committee of the Association was appointed early in 1889 to carry out the organisation of an effective street ambulance for the Metropolis. The labours of the committee were immensely facilitated by the generosity of Mr Bischoffsheim, who placed at its disposal the funds needed to put the scheme in full operation. The Street Ambulance Branch of the Hospitals Association, of which Mr. Bischoffsheim is honorary treasurer and Mr. Ryan honorary secretary, has done a work of genuine philanthropy for London. It has been done so unobtrusively that one hesitates to speak of it in the terms that it deserves; but the fact that 60 ambulance stations, where litters and all "first aid" appliances are kept, have been established throughout London, and that during the year 1892 1,267 cases were removed to hospital on the conveyances of the Street Ambulance Service speak eloquently enough for themselves. It should not be omitted to be stated that Mr. Bischoffsheim has now consented to supply the whole funds for the maintenance of the service as he did for its establishment.

Although the meeting was in the main a business meeting, and the attendance was not large, one could not be present at it, nor can one become acquainted with the work of the Hospitals Association, without feeling very strongly that although the Association does not advertise itself, nor is it ever unduly assertive, it fills a useful and honourable place in the great work of British medical charity. A Central Board of Hospitals for London may or may not be in the future; but until such a Board is organized, it is difficult to see how the Hospitals Association, with its common platform for the discussion of all hospital questions, with its well-informed zeal in promoting all hospital interests, and with its active philanthropy, as evidenced in the Royal National Pension Fund for Nurses and the London Street Ambulance Service, can ever be dispensed with. The Association has won its way with the hospitals by steady helpful work. That position it will no doubt maintain and strengthen in the future, unless or until the interest of the hospitals or the establishment of a new authority like the Central Board, relieves its managers of the responsible duties which they at present so self denyingly carry out in the best interests of the medical institutions of this country.

